# Pre-hospital emergent intubation in trauma patients: the influence of etomidate on mortality, morbidity and healthcare resource utilization

**DOI:** 10.1186/s13049-019-0637-z

**Published:** 2019-06-07

**Authors:** Michael Gäßler, Matthias Ruppert, Rolf Lefering, Bertil Bouillon, Arasch Wafaisade, TraumaRegister DGU

**Affiliations:** 1 Department of Medicine, ADAC Air Rescue Service, Hansastr 19, D-80686 Munich, Germany; 2 Institute for Research in Operative Medicine (IFOM), Witten/Herdecke University, Ostmerheimerstr 200, D-51109 Cologne, Germany; 3 Department of Traumatology and Orthopaedic Surgery, Cologne-Merheim Medical Centre (CMMC), Witten/Herdecke University, Campus Cologne-Merheim, Ostmerheimerstr 200, D-51109 Cologne, Germany; 4 Committee on Emergency Medicine, Intensive Care and Trauma Management (Sektion NIS) of the German Trauma Society (DGU), Cologne, Germany

**Keywords:** Anesthetics i.v., Etomidate, Morbidity, Mortality, Healthcare resource utilization, Trauma

## Abstract

**Background:**

Due to its favorable hemodynamic characteristics and by providing good intubation conditions etomidate is often used for induction of general anesthesia in trauma patients. It has been linked to temporary adrenal cortical dysfunction. The clinical relevance of this finding after a single-dose is still lacking appropriate evidence.

**Methods:**

This retrospective multi-centre study is based on merged data from a German Helicopter Emergency Medical Service (HEMS) database and a large trauma patient registry. All trauma patients who were intubated prior to hospital admission with a documented Injury Severity Score ≥ 9 between 2008 and 2012 were eligible for analysis. The primary endpoint was hospital mortality. Other outcome measures were organ failures, sepsis, length of ventilation, as well as length of stay in hospital and ICU.

**Results:**

One thousand six hundred ninety seven patients were enrolled into the study. Seven hundred sixty two patients received etomidate and 935 patients received other induction agents. The in-hospital mortality was similar in both groups (18.9% versus 18.2%; *p* = 0.71). Incidences of organ failures and sepsis were not increased in the etomidate group. However, health care resource utilization parameters were prolonged (after adjusting: + 1.3 days for ICU length of stay, *p* = 0.062; + 0.8 days for length of ventilation, *p* = 0.15; + 2,7 days for hospital length of stay, *p* = 0.034). A multivariable logistic regression analysis did not identify etomidate as an independent predictor of hospital mortality (OR: 1.10, 95% CI: 0.77–1.57; *p* = 0.60).

**Conclusions:**

This is the largest trial investigating outcome data for trauma patients who had received a single-dose of etomidate for induction of anesthesia. The use of etomidate did not affect mortality. The influence on morbidity and health care resource utilization remains unclear.

## Introduction

Etomidate is a carboxylated imidazole used for induction of general anesthesia and sedation. It was introduced into clinical practice in 1972. Due to its favorable hemodynamic characteristics and by providing good intubation conditions, it became one of the most frequently used drugs for rapid-sequence induction (RSI) of anesthesia in critically ill patients [1–3]. As many trauma patients suffer from hemorrhagic shock, etomidate is often considered the drug of choice during emergent intubation in the field or in the Emergency Department [2].

In the early 1980s first reports of an increased mortality among ventilated trauma patients in the ICU receiving etomidate for prolonged sedation were published [4]. Adrenal cortical inhibition by etomidate was identified as a trigger for adrenal insufficiency in these patients [5–7]. Subsequently, the use of etomidate for prolonged sedation was stopped.

The debate on the impact of a single-dose of etomidate and its clinical relevance in the development of adrenal insufficiency is ongoing ever since [1, 8–12]. In particular with regard to trauma patients we are still lacking appropriate evidence to give a definitive answer to this question. However, the German S3 guideline “Polytrauma” advised against the administration of etomidate for RSI in pre-hospital trauma care [13]. Since then the use of etomidate decreased substantially [14]. Subsequently, a continuous increase of ketamine and especially propofol for pre-hospital trauma anesthesia were observed [14]. The frequent use of propofol may reflect the large in-hospital experience with the drug. On the other hand, a major disadvantage of propofol in the trauma setting is the considerable decrease in mean arterial pressure, which might even be detrimental in hypovolemic patients.

The most suitable anesthetic agent of choice in trauma patients remains undetermined. It has never been proven that a single dose of etomidate in trauma causes increases mortality at all. This led to a remarkable difference in the use of etomidate between countries, regions and even institutions that is still present today. The aim of this study is to address the issue of a single-dose of etomidate in pre-hospital trauma anesthesia by providing outcome data in a large multicentre approach.

## Methods

### Study design and setting

This retrospective study is based on merged data from a Helicopter Emergency Medical Service (HEMS) database (ADAC Air Rescue) and a large trauma patient registry (TraumaRegister DGU®) to investigate the influence of etomidate on mortality and morbidity in trauma patients. The primary endpoint was hospital mortality.

The medical authorities of the ADAC Air Rescue Service and Sektion NIS as the scientific committee of the TraumaRegister DGU® (TR-DGU) approved design and publication of this study. It is in accordance with the publication guidelines of the TraumaRegister DGU® and registered as project ID 2015–050. This study was also approved by the ethics committee of the University of Witten/Herdecke, Faculty of Health (Number 201/2015).

#### ADAC Air Rescue Service

The ADAC Air Rescue Service operates 35 air rescue bases throughout Germany and is therefore one of the largest HEMS providers in Europe. The medical crews consist of an experienced emergency physician and a paramedic (HEMS Technical Crew Member). In 2018 more than 54.000 rescue missions were performed.

A paper and electronic medical record is completed during each mission and for every patient. Information on the pre-hospital course and treatment is documented by the minimal data set for emergency physicians (MIND2). This dataset was established by the German Interdisciplinary Association of Intensive Care and Emergency Medicine (DIVI) and contains basic data on patient characteristics, interventions, and vital signs. In addition to the MIND2 the ADAC Air Rescue Service collects further data including air-rescue specific parameters.

#### TraumaRegister DGU®

The TraumaRegister DGU® of the German Trauma Society (Deutsche Gesellschaft für Unfallchirurgie, DGU) was founded in 1993 [15]. The aim of this multicentre database is a pseudonymised and standardized documentation of severely injured patients. Data are collected prospectively in four consecutive time phases from the site of the accident until dis-charge from hospital: A) Pre-hospital phase, B) Emergency room and initial surgery, C) Intensive care unit and D) Discharge. The documentation includes detailed information on demographics, injury pattern, comorbidities, pre- and in-hospital management, course on intensive care unit, relevant laboratory findings including data on transfusion and outcome of each individual.

The participating hospitals are primarily located in Germany (90%), but a rising number of hospitals of other countries contribute data as well (e.g. Austria, Belgium, Finland, Luxembourg, Slovenia, Switzerland). Currently, approx. 33,000 cases from more than 600 hospitals are entered into the database per year.

The documentation comprises detailed information including standardized scoring systems, e.g. the Injury Severity Score (ISS) [16]. All injuries are coded using the Abbreviated Injury Scale (AIS, version 2005/08) [17]. Organ failure was defined using the Sequential Organ Failure Assessment (SOFA) score [18] where, for each organ, SOFA 3 or 4 was considered as failure. Sepsis was defined according to the criteria of the American College of Chest Physicians/Society of Critical Care Medicine consensus conference definition [19].

### Cohort identification and data collection

In a previous study changes in pre-hospital anesthetic use in trauma patients in the ADAC Air Rescue Service over a ten-year period (2006 to 2015) were investigated. Results have shown an extensive use of etomidate till 2010 (≥ 50% of all anesthesia inductions) followed by a decline in utilization since [14]. Given these results we defined a five-year study period for the current issue to generate a similar patient distribution into two comparable groups.

All trauma patients anesthetized by HEMS physicians between January 1st 2008 and December 31st 2012 were considered for this study. Subsequently HEMS mission data were combined with the TR-DGU register. Data were matched between the two databases using the parameters age, sex, date/time of injury and trauma centre. Both databases did not provide any personal data. This method of anonymized data matching between ADAC Air Rescue and TR-DGU has been practiced successfully before [20].

The HEMS database provided information about the anesthetic agents used, while injury severity and outcome data were taken out of the TR-DGU database. HEMS patients were not assigned to a case in the TR-DGU if: 1) they were admitted to hospitals prior to its participation in the TR-DGU or if the hospital was not participating in the TR-DGU, 2) the patients injuries were finally not severe enough (no requirement of ICU admission) 3) there was no exact matching.

**Fig. 1:**
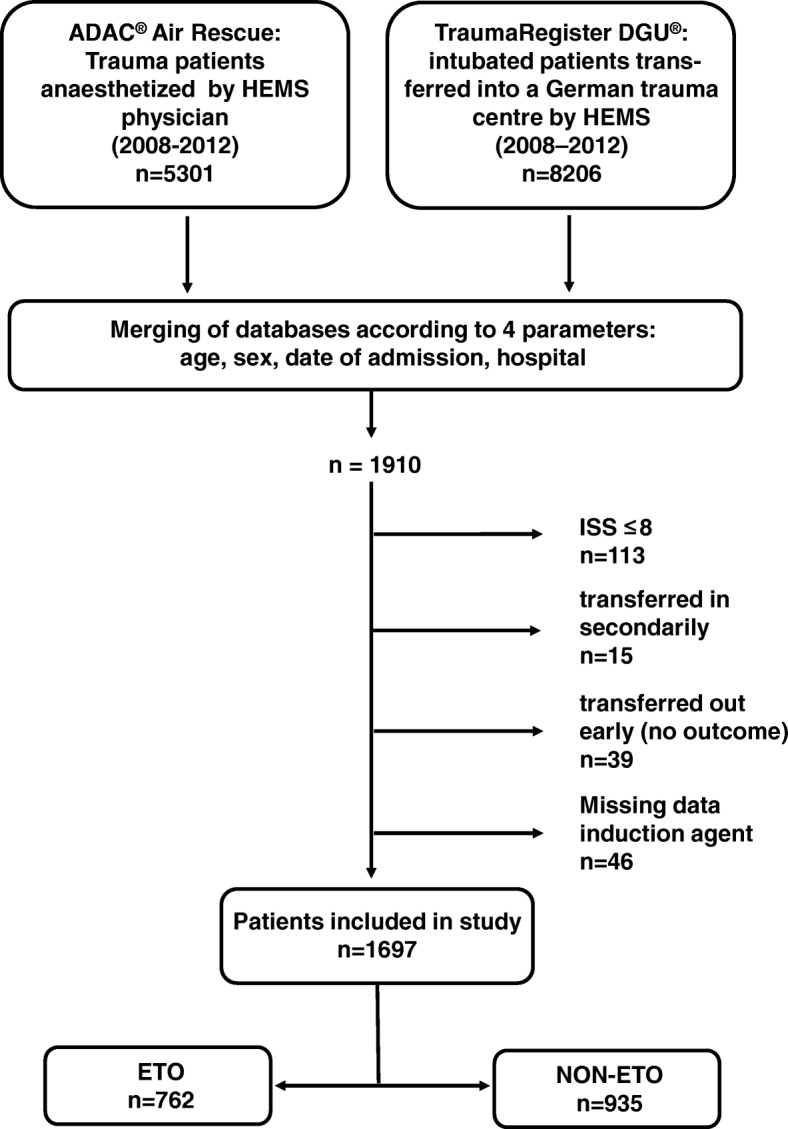
Study outline

A total of 1910 patients out of 5301 patients anesthetized by HEMS physicians were identified as being documented in both databases. The remaining 3391 patients (64%) out of the HEMS database could not be matched with the TR-DGU database. Two hundred thirteen patients were excluded from the study (Fig. 1). Thus 1697 patients were eligible for further analysis and were grouped according the use of etomidate (Fig. 1):

ETO group: Patients who had received etomidate as hypnotic agent for emergent intubation prior to hospital admission (*n* = 762).

NON-ETO group Patients who had received any other induction agent than etomidate prior to hospital admission (*n* = 935).

### Statistical analysis

Data were compared between groups using the Mann-Whitney-U-test for continuous variables and Fischer’s exact test for categorical variables. Data are presented as mean with standard deviation (±SD) for continuous variables and as percentages for counts. In case of skewed distribution, the median is presented in addition. The primary endpoint was hospital mortality.

A multivariable logistic regression analysis with hospital mortality as dependent variable was performed to evaluate the adjusted effect of etomidate where age, sex, unconsciousness (Glasgow Coma Scale ≤8), systolic blood pressure at scene, injury severity, trauma mechanism, hospital level of care (two categories), and year of trauma were the other independent predictors. Length of stay data were adjusted for etomidate, year of trauma, age, and mortality (linear regression). A level of *p* < 0.05 was considered statistically significant. Statistical analysis was performed using SPSS statistical software (SPSS Version 23.0, IBM Inc., Armomk, NY, USA).

## Results

### Study population and baseline characteristics

**Fig. 2:**
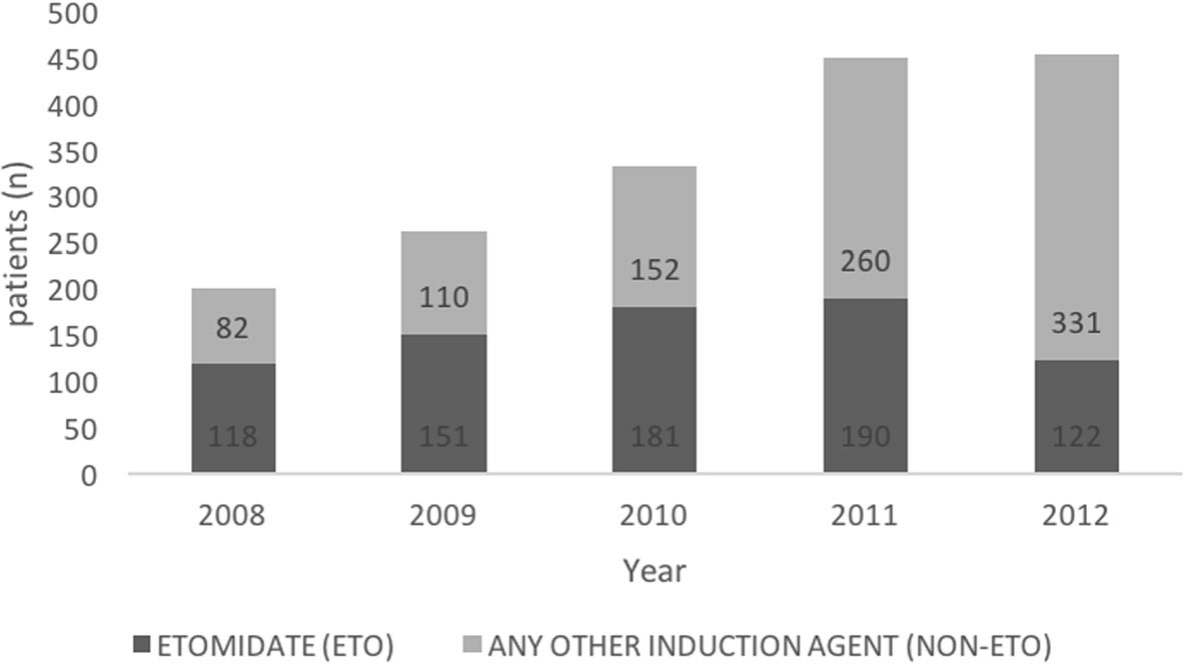
Patient recruitment per year in the ETO and NON-ETO group (*n* = 1697)

We were able to include 1697 patients, out of which 762 patients (44.9%) received etomidate (ETO group) during pre-hospital emergent intubation. Nine hundred thirty five patients received any other intravenous anesthetic (NON-ETO group). Other anesthetics were ketamine, thiopental, propofol, and midazolam. In 2008, 2009, and 2010 more patients received etomidate than any other induction agent (Fig. 2).

Table 1 shows a comparison of demographic and pre-hospital characteristics between the two groups. They were homogeneous for most of the patient characteristics, even though formal statistical differences were observed in age (46 vs. 43 years; *p* < 0.001) and mechanism of injury (93.1% vs. 95.5% blunt trauma; *p* = 0.037). The severity of traumatic injury was comparable in both groups (Table 2).

**Table 1:** Demographic and pre-hospital characteristics of trauma patients in the ETO and NON-ETO group

	ETO	NON-ETO	*p* value
n (total)	**762**	**935**	
Male (n,%)	551/762 (72.3)	711/935 (76.0)	0.083
Traffic accident (n,%)	513/709 (72.4)	647/874 (74.0)	0.46
Blunt trauma (n, %)	663/712 (93.1)	837/876 (95.5)	0.037
SBP scene ≤90 mmHg (n,%)	136/689 (19.7)	178/834 (21.3)	0.45
SBP scene (mmHg: mean ± SD;median;n)	118 ± 34;120 (689)	119 ± 35;120 (834)	0.29
HR scene (1/min: mean ± SD;median;n)	98 ± 24; 99 (458)	99 ± 24; 100 (571)	0.19
GCS (points: mean ± SD;median;n)	10 ± 5; 11 (701)	10 ± 5; 12 (839)	0.97
Age (years: mean ± SD;median;n)	46 ± 20;47 (762)	43 ± 21; 43 (935)	< 0.001

As values were partially missing, the respective population is documented in brackets for continuous variables and in the denominator for categorical variables. SBP = systolic blood pressure; HR = heart rate; GCS = Glasgow Coma Scale

The entries in boldface give the number of patients in the column

**Table 2:** Severity of traumatic injury in the ETO and NON-ETO group

	ETO	NON-ETO	*p* value
n (total)	**762**	**935**	
ISS (points; mean ± SD; median)	27 ± 13; 25	26 ± 13; 25	0.41
AIS_HEAD_ ≥ 3 (%,n)	46.7 (356)	49.4 (462)	0.28
AIS_THORAX_ ≥ 3 (%,n)	57.7 (440)	53.9 (504)	0.12
AIS_ABDOMEN_ ≥ 3 (%,n)	14.6 (111)	15.4 (144)	0.68
AIS_EXTREMITIES_ ≥ 3 (%,n)	44.4 (338)	41.6 (389)	0.26

ISS=Injury Severity Score; AIS = Abbreviated Injury Scale

The entries in boldface give the number of patients in the column

### Mortality

The in-hospital mortality was 18.9% in the ETO group and 18.2% in the NON-ETO group respectively (*p* = 0.71) (Table 3). After adjusting for other predictive variables, the use of etomidate prior to hospital admission was not associated with an increased mortality (OR: 1.10, 95% CI: 0.77–1.57; *p* = 0.60) (Table 4).

**Table 3:** Mortality and length of stay data in the ETO and NON-ETO group

	ETO	NON-ETO	*p* value
n (total)	**762**	**935**	
In-hospital mortality overall (%,n)	18.9 (144)	18.2 (170)	0.71
24-h mortality (%,n)	9.3 (71)	9.4 (88)	1.00
ICU-LOS (days: mean ± SD; median)	12.9 ± 15.2; 8	11.2 ± 13.3; 6	0.002
ICU-LOV (days: mean ± SD; median)	8.0 ± 12.8; 3	6.8 ± 9.7; 2	0.005
HOS-LOS (days: mean ± SD; median)	27.9 ± 28.7; 22	24.7 ± 25.7; 19	0.014

ICU-LOS=Intensive Care unit length of stay; ICU-LOV = Intensive care unit length of ventilation; HOS-LOS=Hospital length of stay

The entries in boldface give the number of patients in the column

**Table 4:** Multivariable logistic regression analysis of risk factors for in-hospital mortality

	OR	95% CI	*p* value
Etomidate	1.10	0,77-1,57	0.60
Age (per year)	1.05	1.04–1.06	< 0.001
Male	0.96	0,66-1,39	0.82
GCS ≤8	3.58	2.55–5.02	< 0.001
SBP scene ≤90 mmHg	2.41	1.67–3.48	< 0.001
ISS (per point)	1.08	1.06–1.09	< 0.001
Blunt trauma	0.47	0.23–0.97	0.04
Hospital level of care	0.57	0.35–0.93	0.03

SBP = systolic blood pressure; GCS = Glasgow Coma Scale; ISS=Injury Severity Score

The entries in boldface give the number of patients in the column

### Morbidity

Cardiovascular failure occurred more frequent in the NON-ETO group (34.7% vs. 43.8%; *p* = 0.004). There were no significant differences in the incidences of other organ failures and sepsis, although for these parameters data were not documented for the entire study population (Table 5).

**Table 5:** Organ failures, sepsis and transfusion in the ETO and NON-ETO group

	ETO	NON-ETO	p value
n (total)	**762**	**935**	
Multiple organ failure (n, %)	175/453 (38.6)	239/548 (43.6)	0.12
Organ failure lung (n, %)	150/453 (33.1)	172/550 (31.3)	0.54
Organ failure coagulopathy (n, %)	99/453 (21.9)	102/550 (18.5)	0.21
Organ failure liver /hepatic (n, %)	15/453 (3.3)	15/550 (2.7)	0.71
Organ failure cardiovascular (n, %)	157/453 (34.7)	241/550 (43.8)	0.004
Organ failure cns (n, %)	149/453 (32.9)	202/550 (36.7)	0.21
Organ failure renal (n, %)	37/453 (8.2)	38/550 (6.9)	0.47
Sepsis (n, %)	44/447 (9.8)	57/540 (10.6)	0.75
pRBC transfusion (n,%)	184/758 (24.3)	222/925 (24.0)	0.91

As values were partially missing, the respective population is documented in brackets for continuous variables and in the denominator for categorical variables. pRBC = packed red blood cells

The entries in boldface give the number of patients in the column

### Healthcare resource utilization

**Fig. 3:**
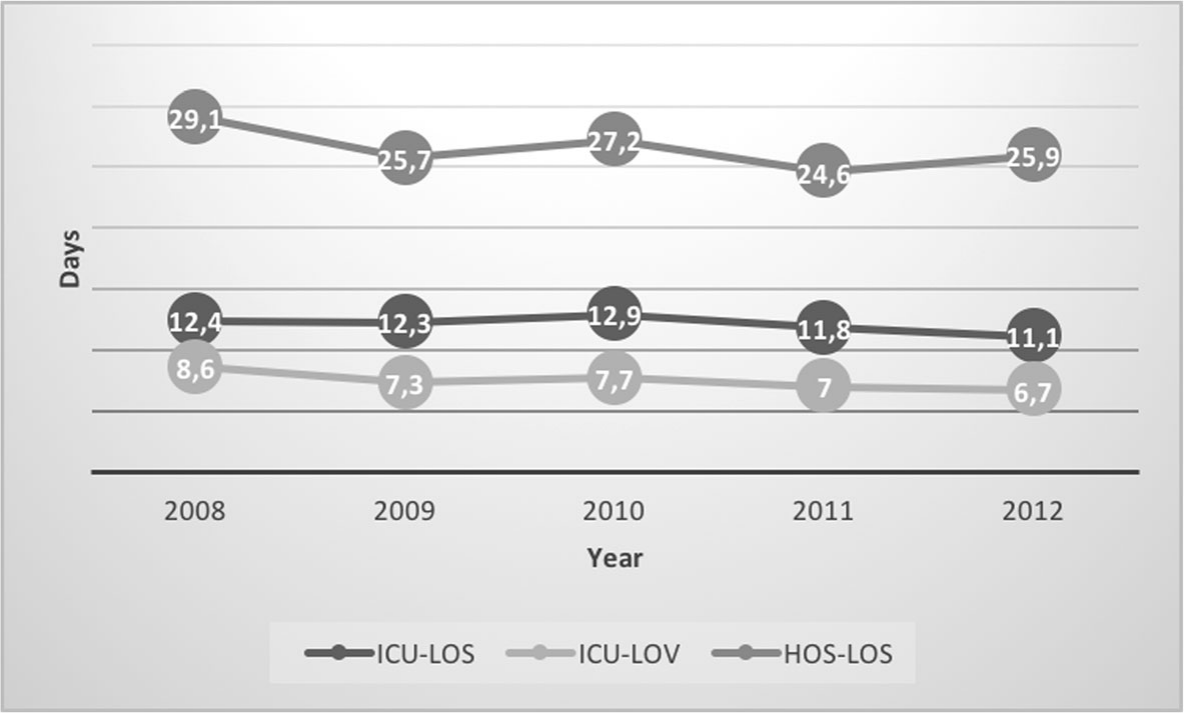
Length of stay and ventilation days per year for the entire study population (n = 1697). ICU-LOS=Intensive Care unit length of stay; ICU-LOV = Intensive care unit length of ventilation; HOS-LOS=Hospital length of stay

Length of stay in the intensive care unit (ICU-LOS), length of mechanical ventilation (ICU-LOV), and length of hospital stay (HOS-LOS) decreased in the entire population over the study period (Fig. 3).

The unadjusted ICU-LOS (12.9 versus 11.2 days; *p* = 0.002), ICU-LOV (8.0 versus 6.8 days; *p* = 0.005) as well as the HOS-LOS (27.9 versus 24.7 days; *p* = 0.014) were prolonged in the ETO group (Table 3). After adjusting for year of trauma, age, and mortality, the effect of etomidate was reduced, but still statistical significant for HOS-LOS (+ 1.3 days for ICU-LOS, *p* = 0.062; + 0.8 days for ICU-LOV, *p* = 0.15; + 2,7 days for HOS-LOS, *p* = 0.034).

## Discussion

To the best of our knowledge, this study is the first attempt to evaluate the outcome of trauma patients who have received etomidate during emergent intubation prior to hospital admission at such a large scale. The ETO and NON-ETO group were comparable in demographic and pre-hospital characteristics. We found no influence of a single dose of etomidate on mortality in trauma patients. We did not find a remarkable difference in morbidity between the two groups, but healthcare resource utilization parameters were prolonged in the ETO group. However, data for organ failures and sepsis were incomplete.

Due to a lack of interfaces between pre-hospital admission and in-hospital patient data documentation, it is difficult to obtain and to merge data from both treatment phases for a large number of patients. The consolidation of the ADAC Air Rescue and the TR-DGU databases gave us the opportunity to generate outcome data for interventions performed in the field prior to hospital admission in a large multicentre approach.

### Mortality

To our knowledge, only four publications have analyzed the mortality after induction of anesthesia in trauma patients using etomidate [21–24]. The largest study we found was a recent retrospective investigation in 968 adult trauma patients, whereof 526 patients were induced in the Emergency Department with etomidate and 442 with ketamine [24]. Hospital mortality was 20.4% for ketamine compared with 17.3% for etomidate (OR: 1.41; 95% CI: 0.92–2.16). In the 4-year study period the use of ketamine compared with etomidate was not associated with an improvement in hospital mortality.

Hinkewich and colleagues [21] investigated 308 trauma patients, in whom induction of anesthesia was performed at a single institution. They observed a remarkable difference in the 28-day mortality of 18.7% for patients receiving etomidate (*n* = 107) and 11.1% for all other anesthetic agents (*n* = 201). However, after multivariate analysis, etomidate could not be identified as an independent predictor of mortality (*p* = 0.11).

A retrospective chart review [22] compared a period of liberal versus a period of limited use of etomidate. Four hundred forty patients were included in the liberal period (58.9% received etomidate) and 882 in the limited period (23.2% received etomidate). The inpatient mortality was similar in the both periods (30% versus 29%; *p* = 0.848).

Jabre and colleagues [23] investigated 469 patients, who received etomidate or ketamine for emergent intubation during a randomized, controlled, single-blind trial. The 28-day mortality as a secondary endpoint was 35% for the etomidate group and 31% for the ketamine group (*p* = 0.36). One hundred four out of the 469 patients had a history of trauma. The 28-day mortality in this subgroup was 30% in the ketamine and 26% in the etomidate group, without revealing a statistical significance. The interpretation of these findings is limited as the cohort is small and there is no further information on ISS, gender or age available.

### Morbidity and healthcare resource utilization

ICU days, ventilator days and hospital stay decreased in general over the past few years. This was also observed in our study population. Etomidate was given predominantly in the first half of the study period and healthcare resource utilization parameters were prolonged in the ETO group. However, after adjusting HOS-LOS was still significantly prolonged in the ETO group.

A trial with a small sample size (*n* = 30) found no significant differences in ICU-LOS, HOS-LOS and ventilator-free days [21]. Equally results without remarkable differences in ICU-LOS and HOS-LOS were reported by comparing a liberal versus a limited period of etomidate application [22]. Upchurch and colleagues did not reveal any distinctions in ICU-LOS and ventilator-free days between patients induced with etomidate or ketamine [24].

In a prospective trial investigating the administration of hypertonic saline administration prior to hospital admission 35 of the 94 patients (37%) received etomidate for emergent intubation in the field [25]. The development of Acute Respiratory Distress Syndrome (ARDS) was defined as primary outcome and was close to reveal statistical significance (*p* = 0.06). In a further stepwise multivariate regression analysis the most severely injured patients were selected. In this small subgroup etomidate was an independent risk factor for the development of ARDS (*p* = 0.02) and Multi Organ Dysfunction Syndrome (MODS) (p = 0.02). In addition, ICU-LOS (p = 0.02), HOS-LOS (p = 0.02), and ICU-LOV (*p* = 0.04) were significant longer in the etomidate group.

Another subgroup analysis out of a previously randomized, controlled trial revealed a greater risk for hospital-acquired pneumonia (HAP) in trauma patients who had received etomidate [26]. One hundred forty nine patients were originally included in the HYPOLITE trial investigating the use of hydrocortisone in patients with severe trauma. All of these patients were part in the following analysis and had received mechanical ventilation for at least 48 h. In a multivariate analysis, etomidate was an independent risk factor for hospital-acquired pneumonia (*p* = 0.016). ICU-LOV did not show a difference between the groups.

Even a lower incidence of cardiovascular failure, defined as any administration of epinephrine/norepinephrine or dopamine use in the clinical course including Emergency Department, was observed in our study for the ETO group. This may reflect the minimal hemodynamic effects of etomidate compared to other induction agents. Even the catecholamine-mediated stabilizing effect of ketamine on the cardiovascular system can fail in patients who are catecholamine depleted [27].

A recent Cochrane review, investigating the effects of a single-dose of etomidate in critically ill patients on mortality, morbidity or healthcare resource utilization, could not reveal any clear answers [8]. However, an increased risk of adrenal gland dysfunction and multi-organ system dysfunction by a small amount was observed. This review was able to include seven studies for the analysis, of which only two studies included traumatized patients. This again demonstrates how limited the available data in this patient population is.

A main limitation for our study is its retrospective nature and the choice of anesthetics at discretion of the emergency physician, not according to a standardized protocol. Data is further limited as exact dosages of induction agents have not been documented in our electronical HEMS database. The NON-ETO group includes patients receiving several different anesthetic agents (thiopental, propofol, ketamine, midazolam). This again reflects the selection of anesthetics at discretion of the emergency physician. All of these drugs are routinely used in pre-hospital trauma anesthesia in Germany. Furthermore, when the two respective databases were merged, out of 5301 patients in the ADAC Air Rescue Service database only 1910 could be assigned to a case in the TR-DGU. A main reason for this is the number of hospitals participating in the TR-DGU over the study period. As shown in Fig. 2 we were able to increase the patient recruitment in each year over the study period due to more hospitals participating in TR-DGU. The documentation of data was incomplete and inconsistent with respect to morbidity (organ failure, sepsis). Also, the cause of death (e.g. bleeding, brain death, organ failure) is not documented in the TR-DGU.

## Conclusions

This is the largest trial investigating outcome data for trauma patients who had received a single-dose of etomidate for induction of anesthesia. The use of etomidate did not affect mortality. The influence on morbidity and health care resource utilization remains unclear. Few trials revealed a higher incidence of lung failure and multi-organ system dysfunction. The present study and one subgroup analysis revealed a prolonged use of healthcare resource utilization. We found no correlation to any organ failure for this result, though data is incomplete with respect to morbidity (organ failure, sepsis). Overall, the available data is limited due to retrospective nature, small and inhomogeneous sample sizes or subgroup analysis.

## Data Availability

Please contact author for data requests.
